# Adaptive fuzzy sliding control of single-phase PV grid-connected inverter

**DOI:** 10.1371/journal.pone.0182916

**Published:** 2017-08-10

**Authors:** Juntao Fei, Yunkai Zhu

**Affiliations:** College of IoT Engineering, Hohai University, Changzhou, China; Chongqing University, CHINA

## Abstract

In this paper, an adaptive fuzzy sliding mode controller is proposed to control a two-stage single-phase photovoltaic (PV) grid-connected inverter. Two key technologies are discussed in the presented PV system. An incremental conductance method with adaptive step is adopted to track the maximum power point (MPP) by controlling the duty cycle of the controllable power switch of the boost DC-DC converter. An adaptive fuzzy sliding mode controller with an integral sliding surface is developed for the grid-connected inverter where a fuzzy system is used to approach the upper bound of the system nonlinearities. The proposed strategy has strong robustness for the sliding mode control can be designed independently and disturbances can be adaptively compensated. Simulation results of a PV grid-connected system verify the effectiveness of the proposed method, demonstrating the satisfactory robustness and performance.

## Introduction

With the depletion of traditional energy sources, the research of energy management [1] [2] and the development of new energy have aroused the interest of researchers. New energy has attracted widespread attention because of its characteristic of being clean and renewable. Sun et.al [3] employed a nonlinear data-enabled predictive energy management strategy for a residential building with PV and battery energy storage using model predictive controller with nonlinear PV and battery models, and a RBF-NN load forecasting algorithm. A key novelty in [3] is to close the gap between building energy management formulations, advanced load forecasting techniques, and nonlinear battery/PV models.

With the development of new energy technologies, PV grid-connected technology has become a hot research topic, more and more people pay attention to the PV power generation. Since PV generation has the characteristics of being intermittent and unstable, higher requirements are put forward about the control of the PV grid-connected inverter. A typical two-stage single-phase PV grid-connected system mainly involves two key technologies: maximum power point tracking (MPPT) and DC-AC inverter control. Common MPPT control method such as constant voltage tracking(CVT)[4], incremental conductance(INC) method [5][6], perturbation and observation method[7], and intelligent methods such as fuzzy control[8], neural network[9][10], particle swarm optimization[11][12] are proposed to track the MPP to increase the efficiency of the PV system. The CVT scheme, which ignores the influence of environment factors, is just a means of voltage stabilizing strategy rather than the MPPT, having low precision and poor adaptability; perturbation and observation method may oscillate in the vicinity of the maximum power point; intelligent methods have satisfactory adaptability to the environment conditions, however the control algorithm is complicated.

Intelligent methods are employed to serve grid-connected inverters. Chen et al. [13] designed a robust fuzzy controller for a PV power inverter with Taguchi tuned scaling factors, easy to be implemented. Logeswaran et al.[14] presented an adaptive neuro-fuzzy model to a multilevel inverter for grid-connected PV system. These strategies are novel to the grid-connected inverter, but there is no discussion about the performance under environmental variations since the PV modules is sensitive to the environment factors such as solar level and environment temperature.

Sliding mode control (SMC)[15] is a nonlinear control method with strong robustness to system uncertainties[16]. Some scholars have employed SMC to control the PV gird-connected inverter [17]. Mohan A et al. [18] presented an adaptive total sliding mode control scheme for the inverter with a full bridge frame and digital phase lock loop (PLL) is used to lock grid frequency and phase, but the system dynamic in the reaching phase is still influenced by uncertainties with the proposed scheme. Kumar N et al.[19] proposed a novel robust and adaptive sliding-mode control for a cascaded two-level inverter-based grid-connected PV system. Dhar S et al.[20] adopted an adaptive finite time fast terminal sliding mode controller for a voltage source converter, steepest ascent MPPT algorithm is presented to track the MPP. However the DC power generated by PV arrays is usually small which need to be enlarged by using a DC-DC inverter. There are rare methods that combine intelligent control and SMC to control the grid-connected inverter.

In this paper, a typical two-stage single-phrase PV grid-connected model is presented. An incremental conductance (INC) method with adaptive step size is used to track the maximum power point. An adaptive fuzzy sliding mode control (AFSMC) scheme which combines both fuzzy logic and SMC is adopted to control the DC-AC inverter to achieve better performance, where the fuzzy system is adopted to estimate the upper bound of the uncertain disturbances caused by the environmental conditions, which could reduce the chattering of the inverter.

This paper has the following characteristics and contributions: The MPPT and DC-AC inverter control are both complemented independently using two different strategies, while most of the existing works only do one of the two key technologies. How an operation of a small scale PV system connected to a micro grid can be achieved is described. Fuzzy control and SMC are combined to control the DC-AC inverter to achieve better performance, the Lyapunov stability theorem based adaptive law guarantees the stability of the PV system. The system has strong robustness for the sliding mode is designed without the information of the system nonlinearities. The using of fuzzy controller enhances the robustness of the system and reduces the chattering. It is convenient to control the inverter for the upper bound of nonlinearities can be adaptively tuned.

This paper is organized as follows: Section 2 presents a model of a two-stage single-phrase PV grid-connected system, an incremental conductance method with adaptive step is given to track the MPP, and an adaptive fuzzy sliding mode strategy is derived to control the DC-AC inverter. Simulation results followed by discussions are presented in section 3 to verify the effectiveness of the proposed control strategies. Finally, conclusions are drawn in section 4.

## Materials and methods

In this section, a two-stage single-phase PV grid-connected inverter model is described and the mathematical expression of the inverter is established, then the INC MPPT scheme is presented to track the MPP of the PV system, the AFSMC strategy is proposed to control the inverter based on Lyapunov theorem.

### Single-phase PV grid-connected model

**Fig 1:**
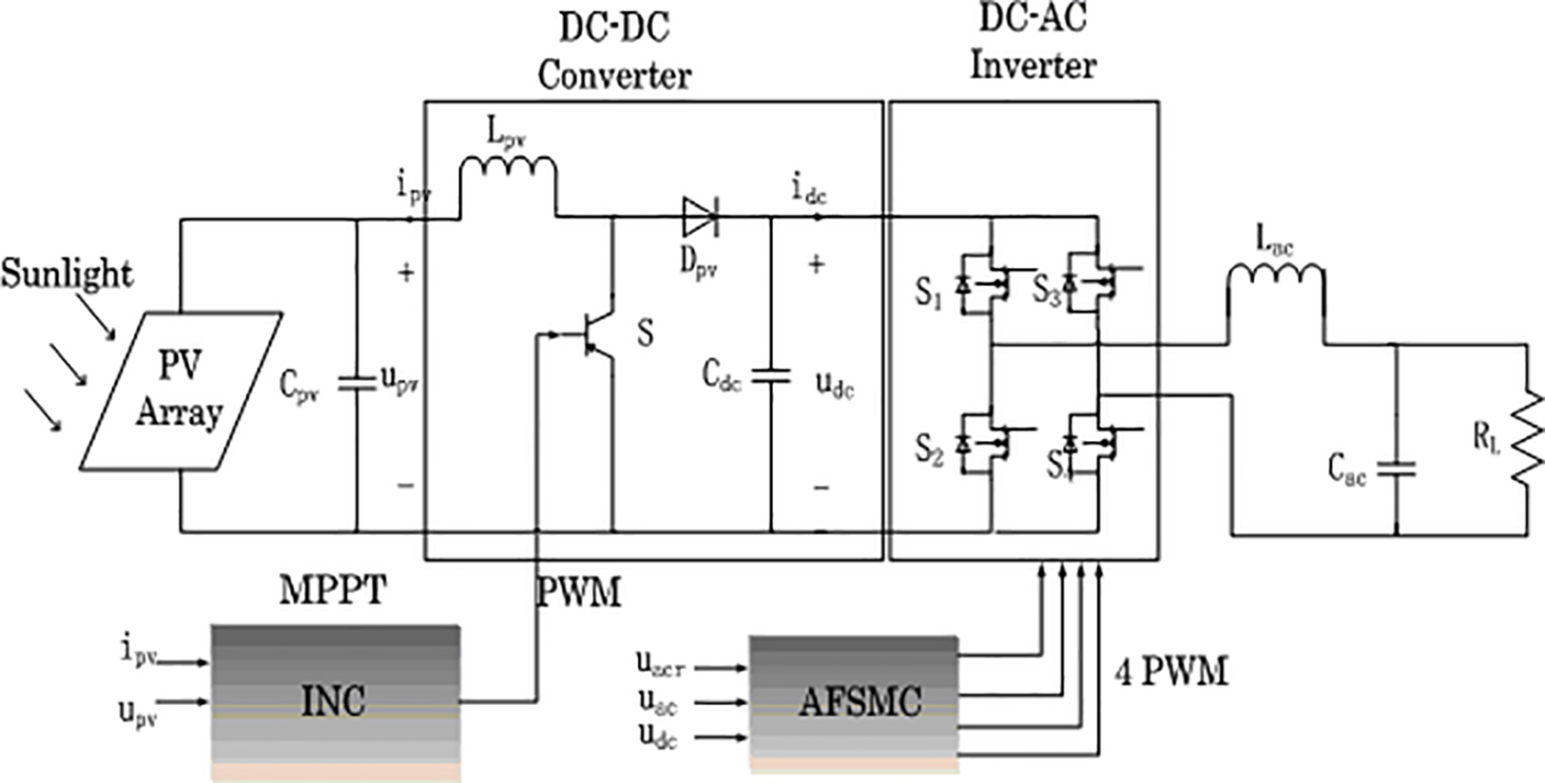
Single-phase PV grid-connected system model.

As shown in Fig 1, a two-stage single-phase PV grid-connected inverter mainly consists of two parts: DC-DC converter and DC-AC inverter. Through the boost DC-DC converter, the PV output voltage can be enlarged and MPPT schemes can be applied. DC-AC inverter turns DC power into AC power with the same frequency and phase with the grid reference voltage.

The boost converter is composed of a controllable power switch *S*_*b*_, inductor *L*_*pv*_, capacitor *C*_*dc*_ and diode *D*_*pv*_. By adjusting the duty cycle of switch *S*_*b*_, the PV system can work at the MPP. The H-bridge DC-AC inverter consists of four controllable power switches. *S*_1_,*S*_4_ and *S*_2_,*S*_3_ form two group arms respectively, by controlling the duty cycle of the two groups of switches, the AC voltage can be obtained. *L*_*ac*_, *C*_*ac*_ are inductor and capacitor at the AC side, *R*_*L*_ is the load.

**Fig 2:**
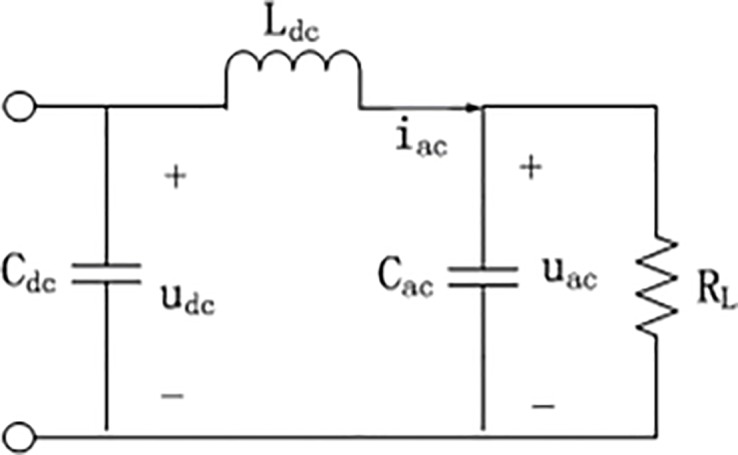
Case while *S*_1_,*S*_4_ are on.

**Fig 3:**
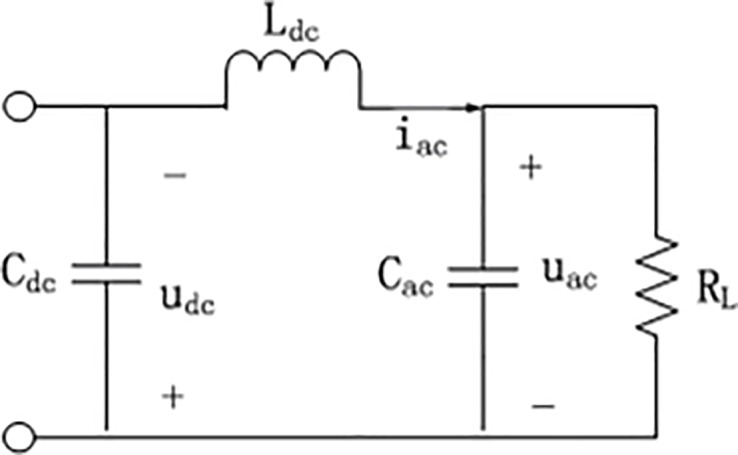
Case while *S*_2_,*S*_3_ are on.

In order to establish the mathematical expression of the inverter, some components need to be idealized. Assuming that *S*_1_−*S*_4_ are all ideal switches with zero on-resistance, whose dead time and capacitance and inductance effect can be ignored, assuming the parasitic resistance of inductor *L*_*ac*_ and capacitor *C*_*ac*_ is small enough. The equivalent circuit when the two groups of switch *S*_1_,*S*_4_ and *S*_2_,*S*_3_ are on is shown in Fig 2 and Fig 3 respectively.

According to according to Kirchhoff's voltage law(KVL) and Kirchhoff's current law(KCL), we have

$$\mathrm{While}\;S_{1},\;S_{4}\;\mathrm{are}\;\mathrm{on}:\;\left\{ \begin{array}{l} -u_{dc}+L_{ac}\frac{di_{ac}}{dt}+u_{ac}=0\\ i_{ac}-\frac{1}{R_{L}}u_{ac}-C_{ac}\frac{du_{ac}}{dt}=0 \end{array}\right.$$ (1)

$$\mathrm{While}\;S_{2},\;S_{3}\;\mathrm{are}\;\mathrm{on}:\;\left\{ \begin{array}{l} u_{dc}+L_{ac}\frac{di_{ac}}{dt}+u_{ac}=0\\ -i_{ac}+\frac{1}{R_{L}}u_{ac}+C_{ac}\frac{du_{ac}}{dt}=0 \end{array}\right.$$ (2)

Assuming that D is the duty cycle of S_1_ and S_4_, then the duty cycle of S_2_ and S_3_ is 1-D. Combining (1) and (2), the mathematical expression of the inverter can be described as (3)

$$\{\begin{array}{l} L_{ac}\frac{di_{ac}}{dt}=(2D-1)u_{dc}-u_{ac}\\ C_{ac}\frac{du_{ac}}{dt}=i_{ac}-\frac{1}{R_{L}}u_{ac} \end{array}$$ (3)

After elimination and consolidation, the dynamic equation of the inverter is derived as (4)

$$\frac{d^{2}u_{ac}}{dt^{2}}=-\frac{1}{R_{L}C_{ac}}\frac{du_{ac}}{dt}-\frac{1}{L_{ac}C_{ac}}u_{ac}+\frac{2D-1}{L_{ac}C_{ac}}u_{dc}$$ (4)

Since *u*_*ac*_ and its derivative as well as the DC side voltage *u*_*dc*_ can be measured and provided to the controller, the Eq (4) has practical significance. In practical applications, the inverter is affected by parameter variations and external disturbances such as environmental variations, as a result, the expression of (4) cannot represent the model of real inverter any longer, so (4) need to be modified. Considering the nonlinearities in the inverter model, then the mathematical expression of the inverter is obtained as (5)

$$\frac{d^{2}u_{ac}}{dt^{2}}=-\frac{1}{R_{L}C_{ac}}\frac{du_{ac}}{dt}-\frac{1}{L_{ac}C_{ac}}u_{ac}+\frac{2D-1}{L_{ac}C_{ac}}u_{dc}+g(t)$$ (5)

where *g*(*t*) is the system unknown nonlinearities.

### MPPT strategy

**Fig 4:**
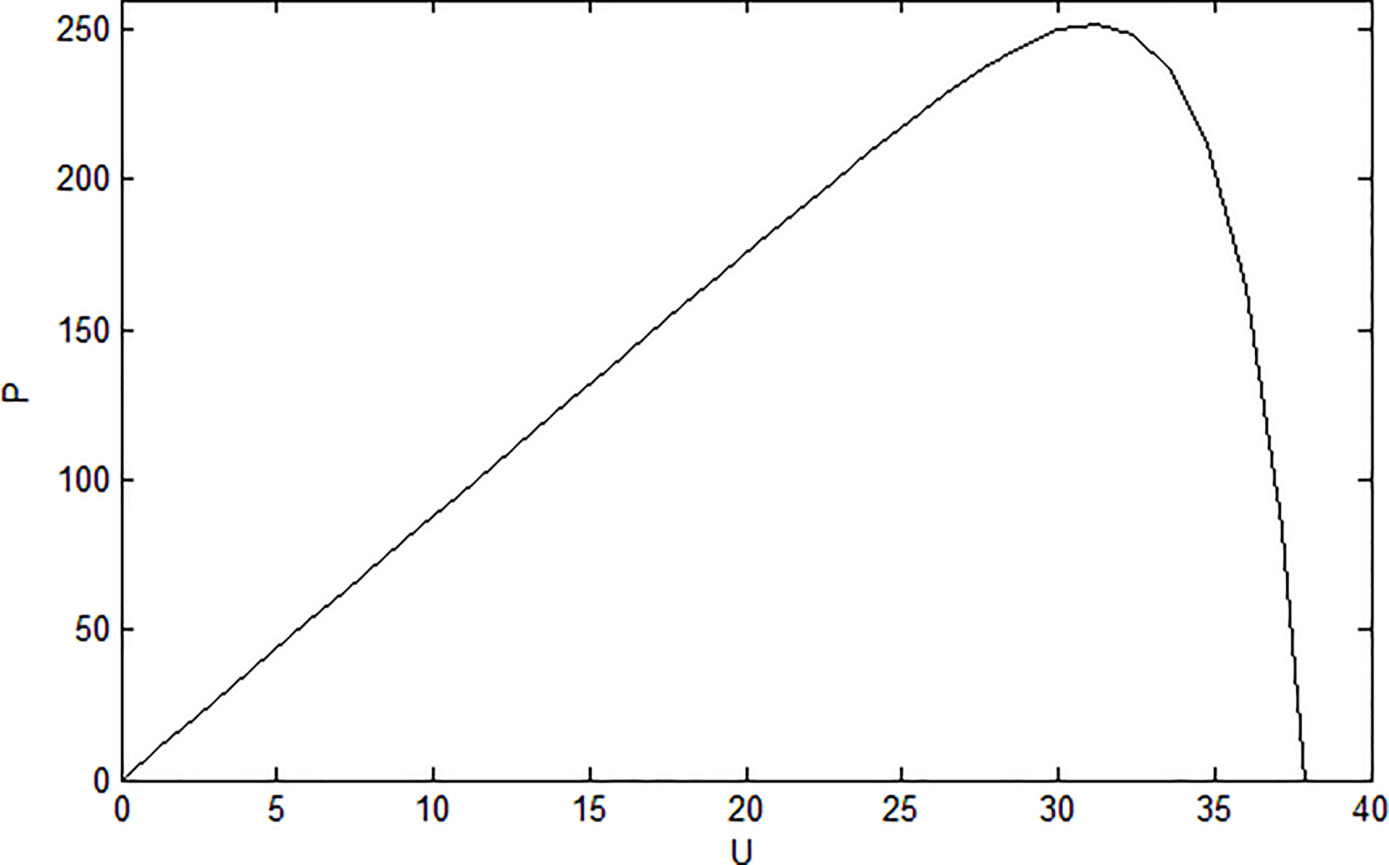
P-U characteristic of PV module.

In practical applications, the PV module is sensitive to the environment factors, the MPPT is needed to improve the efficiency of the PV system. In order to track the MPP, it is necessary to study the characteristics of PV arrays. The P-U characteristic of PV module is shown in Fig 4.

It can be concluded that at the MPP, we have

$$\frac{dP}{dU_{pv}}=I_{pv}+U_{pv}\frac{dI_{pv}}{dU_{pv}}=0$$ (6)

$$\mathrm{Rewriting}\;\left(6\right)\;\mathrm{as}\;\frac{dI_{pv}}{dU_{pv}}=-\frac{I_{pv}}{U_{pv}}$$ (7)

The essence of searching the MPP by the INC scheme is to search the working point that satisfies the Eq (7).In numerical algorithm, *dI*_*pv*_ and *dU*_*pv*_ can be rewritten as

$$\left\{ \begin{array}{l} dI_{pv}=\Delta I_{pv}=I_{pv}(k)-I_{pv}(k-1)\\ dU_{pv}=\Delta U_{pv}=U_{pv}(k)-U_{pv}(k-1) \end{array}\right.$$ (8)

In PV system, the boost converter satisfies the relationship: *U*_*pv*_ = (1−*D*_*b*_)*U*_*dc*_, where *D*_*b*_ is the duty cycle of the switch *S*_*b*_. When *U*_*dc*_ keeps constant, the PV output voltage *U*_*pv*_ and *D*_*b*_ vary conversely. Hence we can get the suitable *U*_*pv*_ which makes the PV system working at the MPP by adjusting *D*_*b*_.

From the P-U characteristic, there are three situations in the process of MPPT: when *dI*_*pv*_/*dU*_*pv*_ > −*I*_*pv*_/*U*_*pv*_, that is *dP*/*dU*_*pv*_ > 0, the current working point locates on the left side of the MPP, we need to reduce the *D*_*b*_ to increase *U*_*pv*_. When *dI*_*pv*_/*dU*_*pv*_ < −*I*_*pv*_/*U*_*pv*_, that is *dP*/*dU*_*pv*_ < 0, the current working point locates on the right side of the MPP, we need to increase *D*_*b*_ to decrease *U*_*pv*_. When *dI*_*pv*_/*dU*_*pv*_ = −*I*_*pv*_/*U*_*pv*_, that is *dP*/*dU*_*pv*_ = 0, the current working point is the MPP.

Choosing adaptive step size as $\lambda \times \left|\frac{dP}{dU_{pv}}\right|$, where *λ* is a positive constant, the iteration algorithm is expressed as (9)

$$D_{b}(k)=D_{b}(k-1)\pm \lambda \left|\frac{dP}{dU_{pv}}\right|$$ (9)

**Fig 5:**
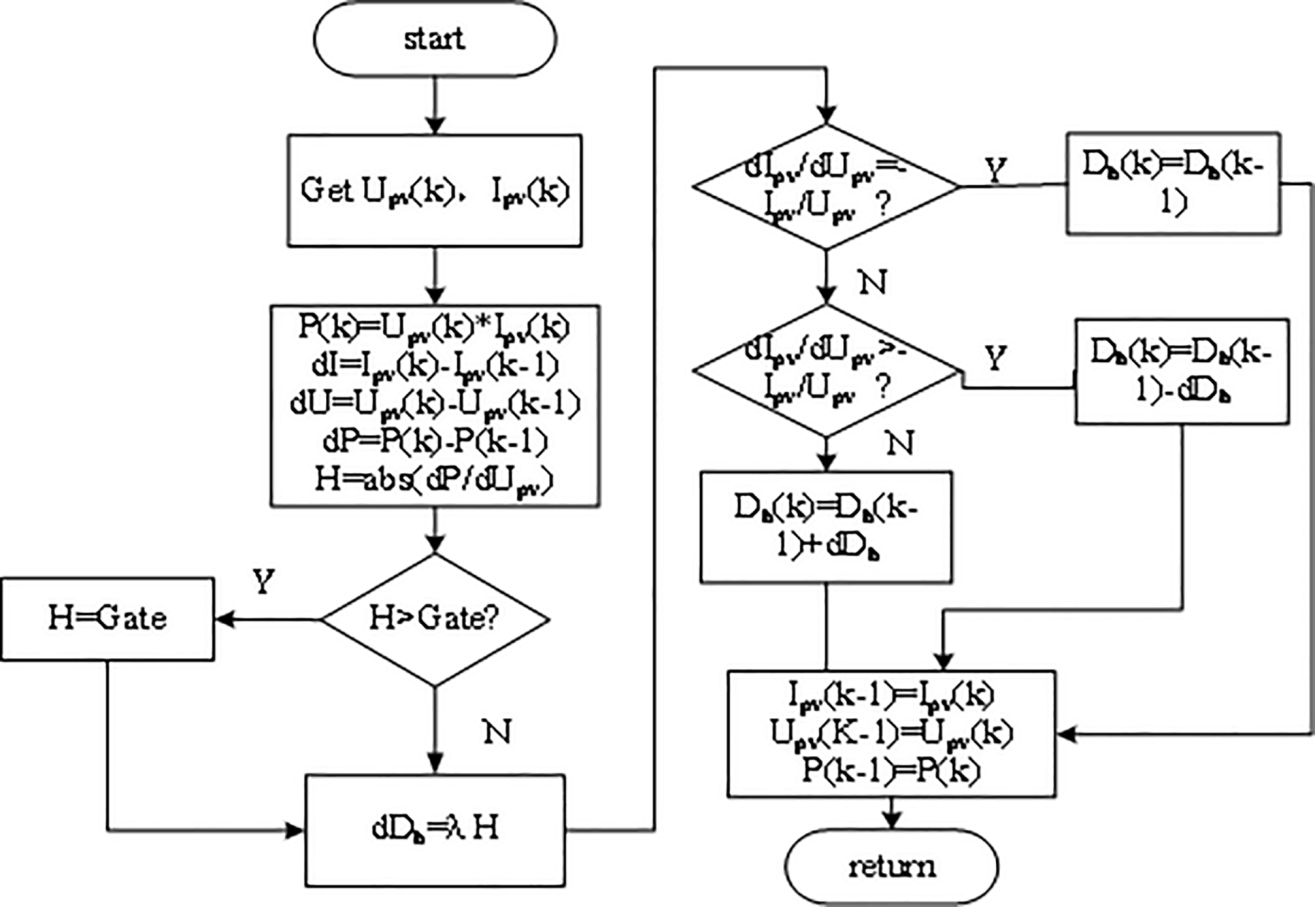
INC method with adaptive step size.

The sign in Eq (9) is determined by the working point of the PV system. Detailed processes are shown in Fig 5 where Gate is a threshold parameter to prevent step too large step.

## Design of adaptive fuzzy sliding mode control

SMC is a special nonlinear control method with notable robustness in disturbance rejection because the sliding mode can be designed without the information of nonlinearities. Grid-connected inverter is a tracking system, whose goal is to track the grid reference voltage.

**Fig 6:**
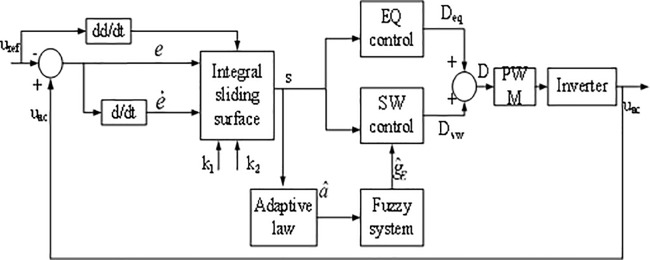
Block diagram of AFSMC.

The AFSMC algorithm is shown in Fig 6. Firstly, select an integral sliding surface, then calculate the equivalent (EQ) control law by setting $\dot{s}=0$ without considering the nonlinearities. After that, employ a switching (SW) control law to compensate the unknown nonlinearities. Finally, adopt a fuzzy system to estimate the upper bound of the nonlinearities.

### Sliding mode control

The first step of SMC is to select a sliding surface, then design the control law to force the system state trajectories toward the sliding surface and stay on it.

Choosing an integral sliding surface as

$$s(t)={\dot{u}}_{ac}(t)-\int_{0}^{t}[{\ddot{u}}_{ref}(t)-k_{1}\dot{e}(t)-k_{2}e(t)]dt$$ (10)

where *u*_*ref*_ is the grid reference voltage, *e* = *u*_*ac*_−*u*_*ref*_ is the tracking error between *u*_*ac*_ and *u*_*ref*_, *k*_1_ and *k*_2_ are positive constants.

The derivative of sliding surface is derived as

$$\begin{array}{l} \dot{s}={\ddot{u}}_{ac}-{\ddot{u}}_{ref}+k_{1}\dot{e}+k_{2}e\\ =-\frac{1}{R_{L}C_{ac}}{\dot{u}}_{ac}-\frac{1}{L_{ac}C_{ac}}u_{ac}+\frac{2D-1}{L_{ac}C_{ac}}u_{dc}+g-{\ddot{u}}_{ref}+k_{1}\dot{e}+k_{2}e \end{array}$$ (11)

Setting $\dot{s}=0$ and ignoring the nonlinearities, we get the equivalent control *D*_*eq*_ as

$$D_{eq}=0.5[1+\frac{L_{ac}C_{ac}}{u_{dc}}(\frac{1}{R_{L}C_{ac}}{\ddot{u}}_{ac}+\frac{1}{L_{ac}C_{ac}}u_{ac}+{\ddot{u}}_{ref}-k_{1}\dot{e}-k_{2}e)]$$ (12)

where $\dot{s}=0$ can be satisfied by choosing suitable *k*_1_ and *k*_2_.

However, the control law described by (12) cannot be implemented directly because the nonlinearities are ignored. Redesigning the control law as

$$D=D_{eq}+D_{sw}$$ (13)

$$D_{sw}=\text{-}0.5\frac{L_{ac}C_{ac}}{u_{dc}}g_{E}\mathrm{sgn}(s)$$ (14)

where |*g*| < *g*_*E*_, *g*_*E*_ is the upper bound of system nonlinearities.The switching control law is adopted to compensate the unknown nonlinearities to ensure the sliding condition can be always satisfied.

### Adaptive fuzzy Sliding mode control

In fact, the upper bound of the system nonlinearities is difficult to be measured in practical applications, if an empirical value is selected, then too large value may result in large oscillation, while too small one will not be able to compensate the nonlinearities. So a fuzzy system is proposed to approach the optimal upper bound of the nonlinearities adaptively.

Choosing the tracking error *e* as the input and the upper bound of uncertainties *g*_*E*_ as the output to form a single input and single output fuzzy system with the fuzzy rules as follows:

$$\mathrm{Rule}\;i:\;\mathrm{If}\;e\;\mathrm{is}\;F_{e}^{i},\;\mathrm{then}\;g_{E}\;\mathrm{is}\;a_{i}$$

where $F_{e}^{i}$ (i = 1,2,3……m) and *a*_*i*_ (i = 1,2,3…….m) belong to the fuzzy input and output set respectively, and m is the number of fuzzy rules. The defuzzification of the fuzzy output is accomplished by the method of center-of -gravity determined by (15)

$$g_{E}=\frac{\sum w_{i}\times a_{i}}{\sum w_{i}}=a^{T}\xi$$ (15)

where *a* is an adjustable parameter vector, *ξ* is a fuzzy basis function vector, and $\xi_{i}=\frac{w_{i}}{\sum w_{i}}$, i = 1,2,3…..m.

According to the universal approximation theory, there exists an optimal parameter *a* that satisfied $g_{E}^{*}=g_{E}+\varepsilon =a^{*}{}^{T}\xi$, where *ε* is the approximation error bounded by |*ε*| < *E*, *E* is a positive constant.

Employing an adaptive fuzzy control system to approximate the upper bound of system nonlinearities *g*_*E*_ is described as (16)

$${\hat{g}}_{E}={\hat{a}}^{T}\xi$$ (16)

where $\hat{a}$ is the estimation of a*.

Applying the fuzzy system (16) to estimate *g*_*E*_ in the switching term described by (14), replace *g*_*E*_ in (14) by ${\hat{g}}_{E}$, we get the new switching control law as (17)

$$D_{sw}=\text{-}0.5*\frac{L_{ac}C_{ac}}{u_{dc}}{\hat{g}}_{E}\mathrm{sgn}(s)$$ (17)

Combining (12), (13) and (17) yields

$$D=0.5[1+\frac{L_{ac}C_{ac}}{u_{dc}}(\frac{1}{R_{L}C_{ac}}{\ddot{u}}_{ac}+\frac{1}{L_{ac}C_{ac}}u_{ac}+{\ddot{u}}_{ref}-k_{1}\dot{e}-k_{2}e-{\hat{g}}_{E}\mathrm{sgn}(s))]$$ (18)

Taking derivative of the sliding function (10) and applying the control law (18), the following equation can be obtained

$$\begin{array}{l} \dot{s}={\ddot{u}}_{ac}-{\ddot{u}}_{ref}+k_{1}\dot{e}+k_{2}e\\ =-\frac{1}{R_{L}C_{ac}}{\dot{u}}_{ac}-\frac{1}{L_{ac}C_{ac}}u_{ac}+\frac{2D-1}{L_{ac}C_{ac}}u_{dc}+g-{\ddot{u}}_{ref}+k_{1}\dot{e}+k_{2}e\\ =g-{\hat{g}}_{E}\mathrm{sgn}(s) \end{array}$$ (19)

Define $\tilde{a}=\hat{a}-a^{*}$ as the error between a* and its estimation $\hat{a}$.

Select a Lyapunov function candidate as

$$V=\frac{1}{2}s^{2}+\frac{1}{2\eta }{\tilde{a}}^{T}\tilde{a}$$ (20)

where *η* is a positive constant.

Differentiating (20) with respect to time derives

$$\begin{array}{l} \dot{V}=s\dot{s}+\frac{1}{\eta }{\tilde{a}}^{T}\dot{\tilde{a}}=s(g-{\hat{g}}_{E}\mathrm{sgn}(s))+\frac{1}{\eta }{\tilde{a}}^{T}\dot{\tilde{a}}\\ \;=s(g-{\hat{a}}^{T}\xi \mathrm{sgn}(s))+\frac{1}{\eta }{\tilde{a}}^{T}\dot{\tilde{a}}=s(g-{\hat{a}}^{T}\xi \mathrm{sgn}(s))+\frac{1}{\eta }{\left(\hat{a}-a^{*}\right)}^{T}\dot{\tilde{a}}\\ \;=sg-{\hat{a}}^{T}\xi \left|s\right|+\frac{1}{\eta }{\left(\hat{a}-a^{*}\right)}^{T}\dot{\tilde{a}}=sg-{\hat{a}}^{T}\xi \left|s\right|+\frac{1}{\eta }\hat{a}\dot{\tilde{a}}-\frac{1}{\eta }a^{*}{}^{T}\dot{\tilde{a}} \end{array}$$ (21)

To ensure $\dot{V}\leq 0$, the adaptive law is designed as (22)

$$\dot{\hat{a}}=\dot{\tilde{a}}=\eta \left|s\right|\xi$$ (22)

Apply (22) into (21) derives that

$$\begin{array}{l} \dot{V}=sg-\left|s\right|a^{*T}\xi \leq \left|s\right|g-\left|s\right|a^{*T}\xi \\ \;=-(g_{E}-g+\varepsilon )\left|s\right|\leq -(g_{E}-\left|g\right|+\varepsilon )\left|s\right|\\ \;=-(g_{E}-\left|g\right|)\left|s\right|-\varepsilon \left|s\right|\leq -\varepsilon \left|s\right|\\ \;\leq -\left|\varepsilon \right|\left|s\right|\leq 0 \end{array}$$ (23)

$\dot{V}$ is negative semi-definite implies that the system can be asymptotically stable according to Lyapunov stability theorem. Furthermore, *V*(0) and *V*(*t*) are all bounded, according to the Barbalat lemma, it can be concluded that *V* → 0 as *t* → ∞, then the tracking error *e* → 0 as *t* → ∞, which means the output of the inverter can asymptotically track the grid reference voltage with zero steady state error.

## Results and discussions

In order to verify the effectiveness of the proposed control strategies, A PV module with 500W maximum power is employed, a two-stage single-phase PV grid-connected system model is built in Simulink with the parameters shown in Table 1.The following paragraph describes the performance of the inverter under environmental variations and the performance comparison with SMC.

**Table 1:** Simulation parameters.

Parameters	Values	Parameters	values
*L*_*pv*_	3×10^−4^ *H*	*u*_*ref*_	$220\sqrt{2}\sin(100\pi t)/50\mathrm{Hz}$
*C*_*pv*_	10^−3^ *F*	*C*_*ac*_	2.82×10^−5^ *F*
*C*_*dc*_	10^−4^ *F*	*L*_*ac*_	0.048 *H*
*λ*	10^−6^	*η*	10
Gate	60	*k*_1_	8×10^4^
*R*_*L*_	400Ω	*k*_2_	5×10^8^

### Performance of the inverter under environmental variations

In practical applications, the PV module is sensitive to the variations of environmental conditions such as solar level and environment temperature. In order to verify the feasibility of the proposed strategies, the solar radiation and temperature factor are changed in the simulation.

**Fig 7:**
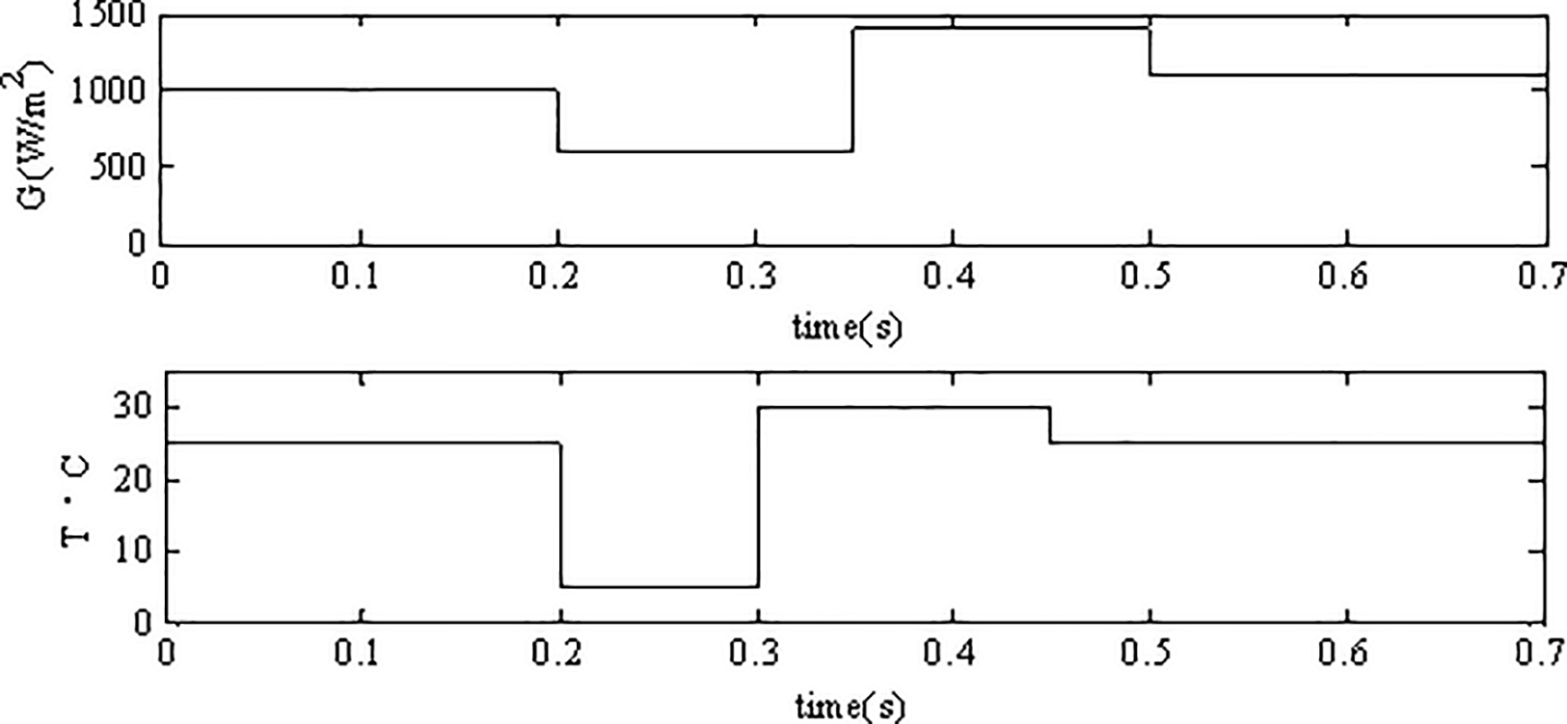
Environmental variations.

As is shown in Fig 7, the Y-label G is the solar radiation, T represents the temperature. the initial solar radiation is set to be 1000 *W*/*m*^2^ (100%), and it decreases to 600 *W*/*m*^2^ (60%) at 0.2s, increases to 1400 *W*/*m*^2^ (140%) at 0.35s then decreases to 1100 *W*/*m*^2^ (110%) at time 0.5s; the initial value of the temperature is set to be 25°C(100%), and it decreases to 5°C(20%) at time 0.2s, decreases to 30°C(120%) at time 0.3s finally it returns to 25°C(100%)at time 0.4s.

**Fig 8:**
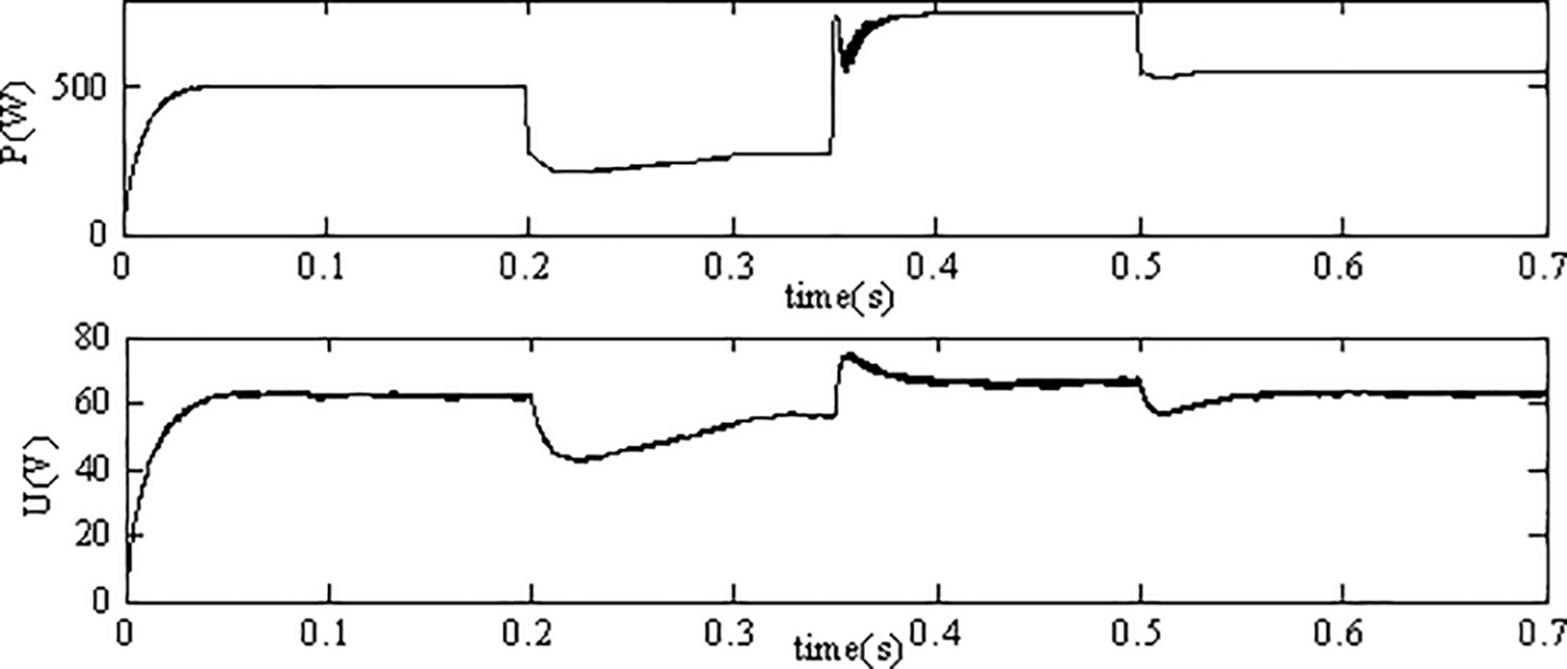
Performance of MPPT.

Fig 8 shows the dynamic processes of the output power and voltage of the PV module under environmental variation condition. The simulation results show that the maximum power of the PV modules is sensitive to the variations of environment conditions especially the solar level, variation tendency of the power is consistent with that of the lighting. The proposed INC MPPT method can be adapted to these variations quickly and track the MPP with less chattering phenomena.

**Fig 9:**
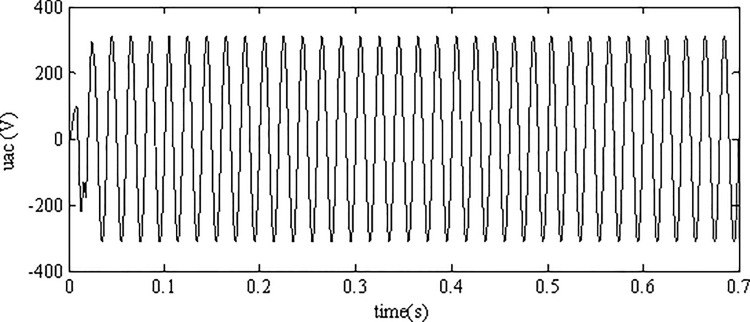
Output voltage of the inverter.

A reliable inverter control scheme should have the ability to keep its output voltage constant with the grid reference voltage under environmental variations. Fig 9 depicts the output voltage of the inverter which is smooth without obvious burr indicating the robustness of the proposed AFSMC strategy.

**Fig 10:**
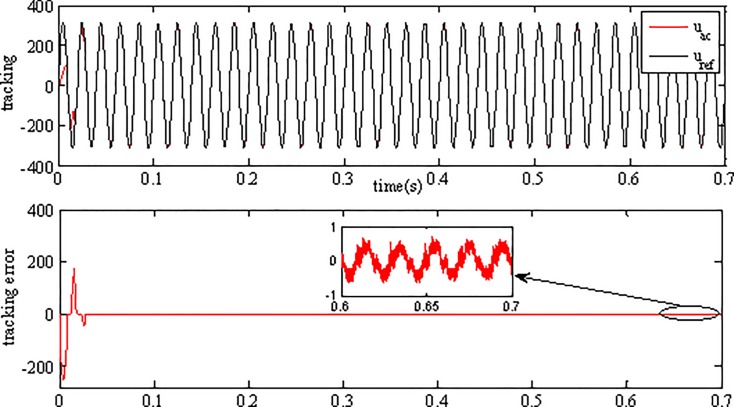
Voltage tracking performance of the inverter.

**Fig 11:**
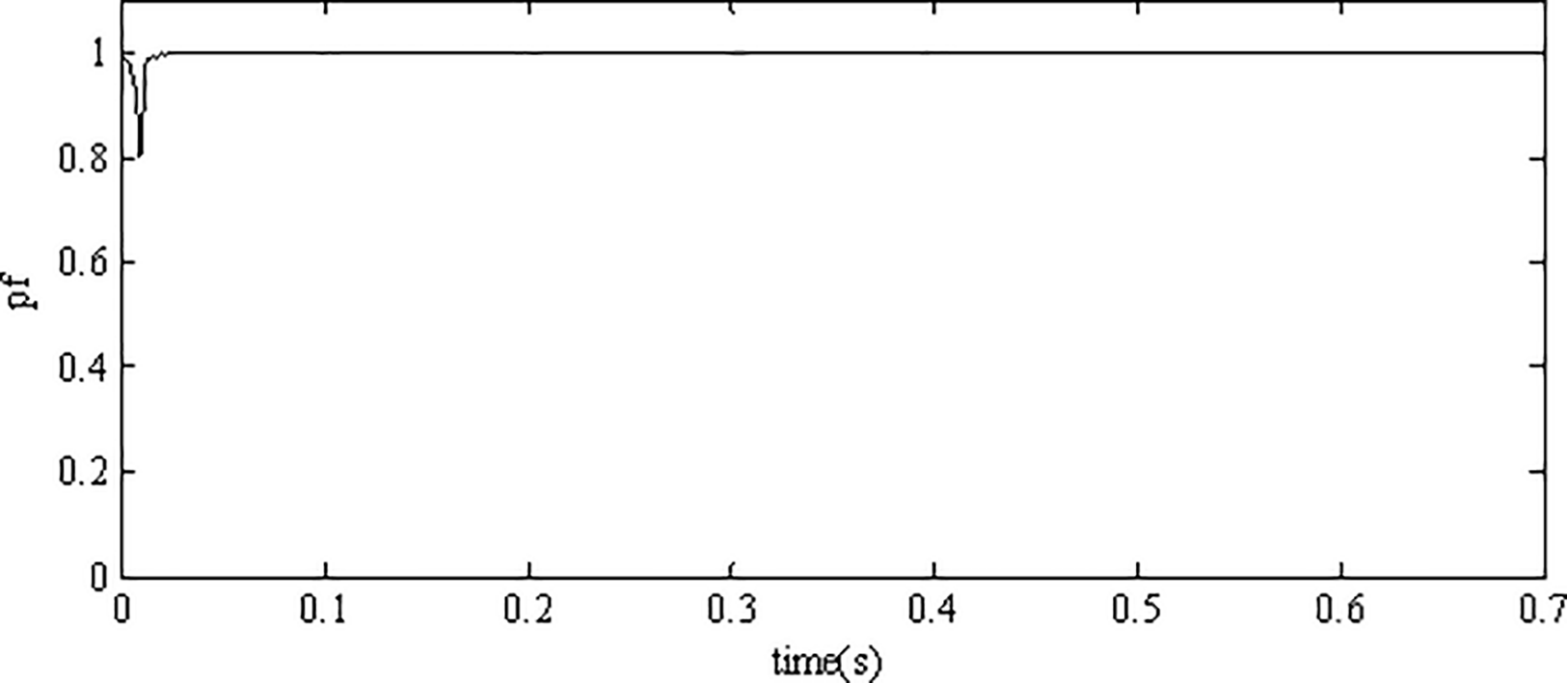
Power factor of the inverter.

Tracking performance in Fig 10 shows that the inverter can track the grid reference voltage in only two cycles. When the irradiance level and surrounding temperature vary, the inverter output voltage can always keep consistent with the reference voltage, showing the robustness to environmental variations. The tracking error is very small, which is only 0.25% of the grid reference voltage in amplitude. Simulation results demonstrate that the inverter using AFSMC scheme is insensitive to the variations of environment factors. Fig 11 depicts the power factor (pf) of the gird-connected inverter, as can be seen, the power factor is close to 1 showing high power quality of proposed strategy.

### Performance comparison with sliding mode control

In order to verify the superiority of proposed AFSMC control strategy, a comparison between AFSMC and SMC is accomplished under the same conditions. Both of the parameters of the two schemes are chosen optimally.

**Fig 12:**
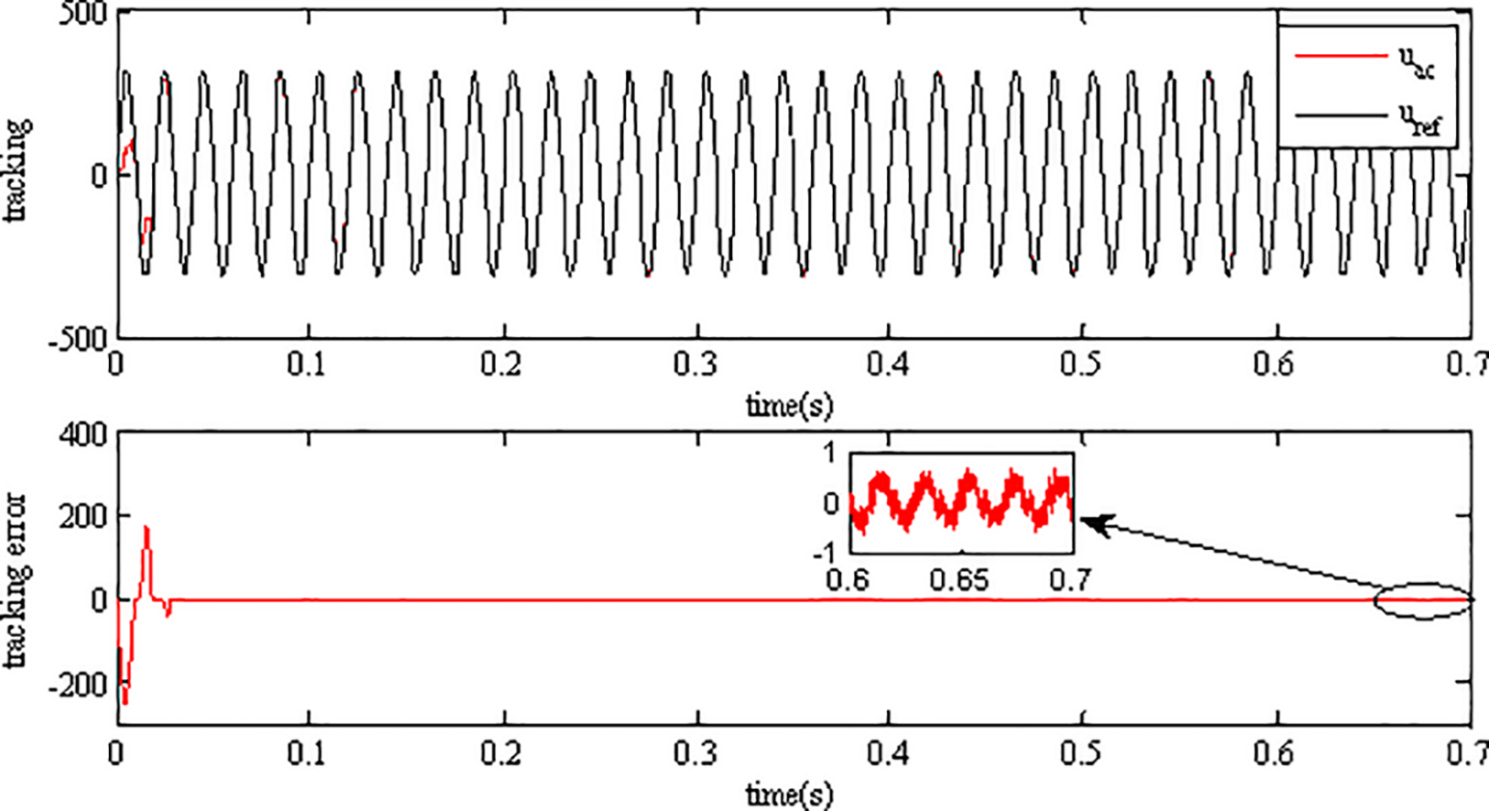
Tracking performance of inverter using AFSMC.

**Fig 13:**
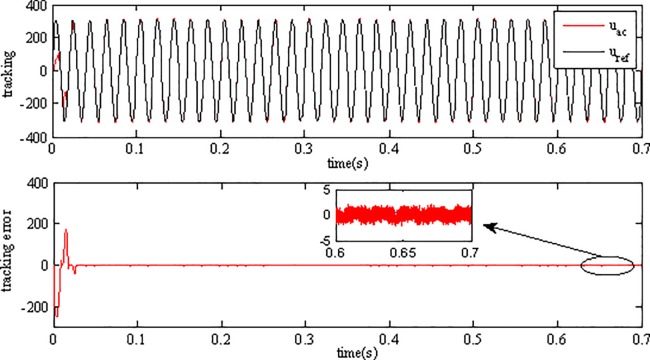
Tracking performance of inverter using SMC.

Fig 12 depicts the performance of voltage tracking of the inverter when using AFSMC method, while the performance of the inverter using SMC scheme is shown in Fig 13. As can be seen, although the output voltage of inverter can track the grid reference voltage and keep consistent with it using both of the two strategies, but the tracking error when using SMC is much bigger than that using AFSMC, indicating that the proposed strategy has better steady performance than SMC.

**Fig 14:**
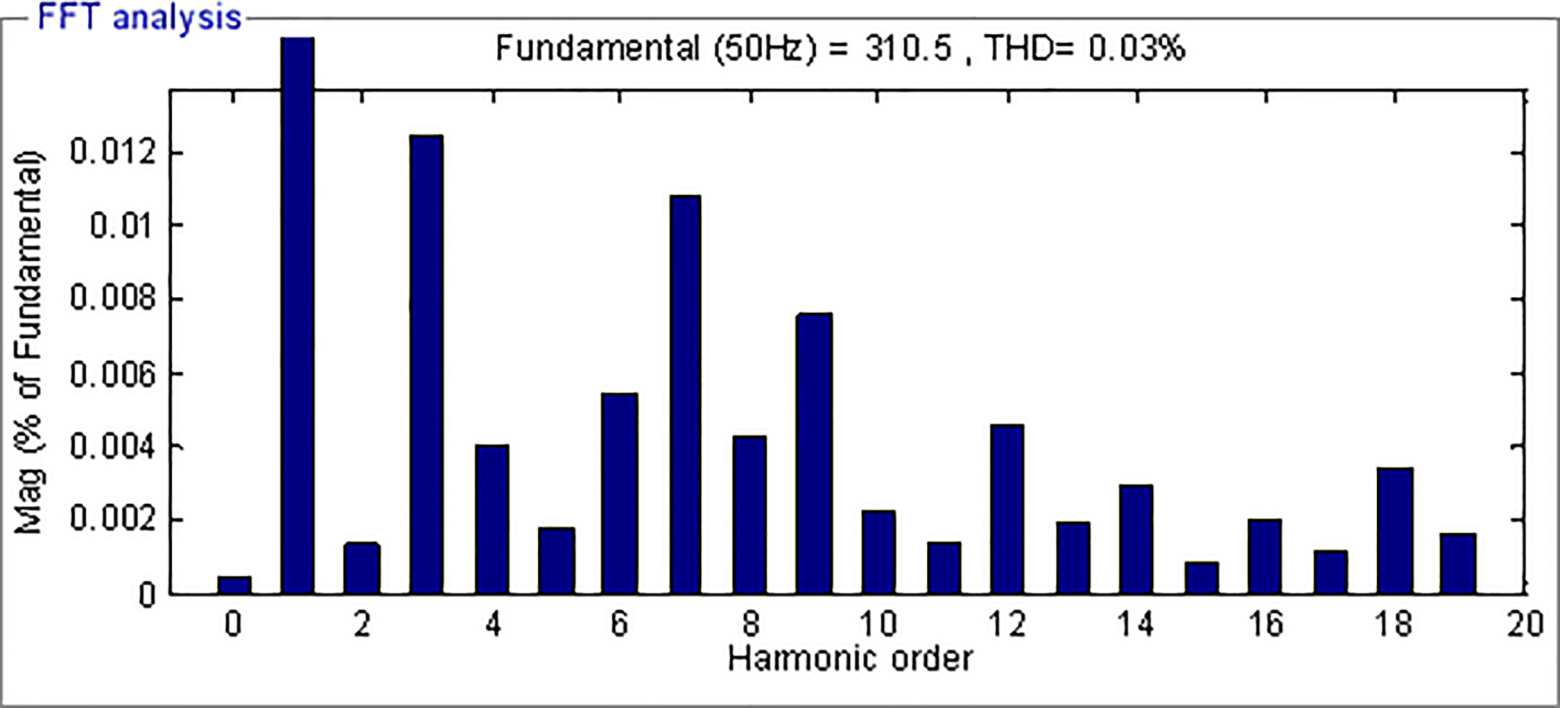
FFT analysis of the output voltage using AFSMC.

**Fig 15:**
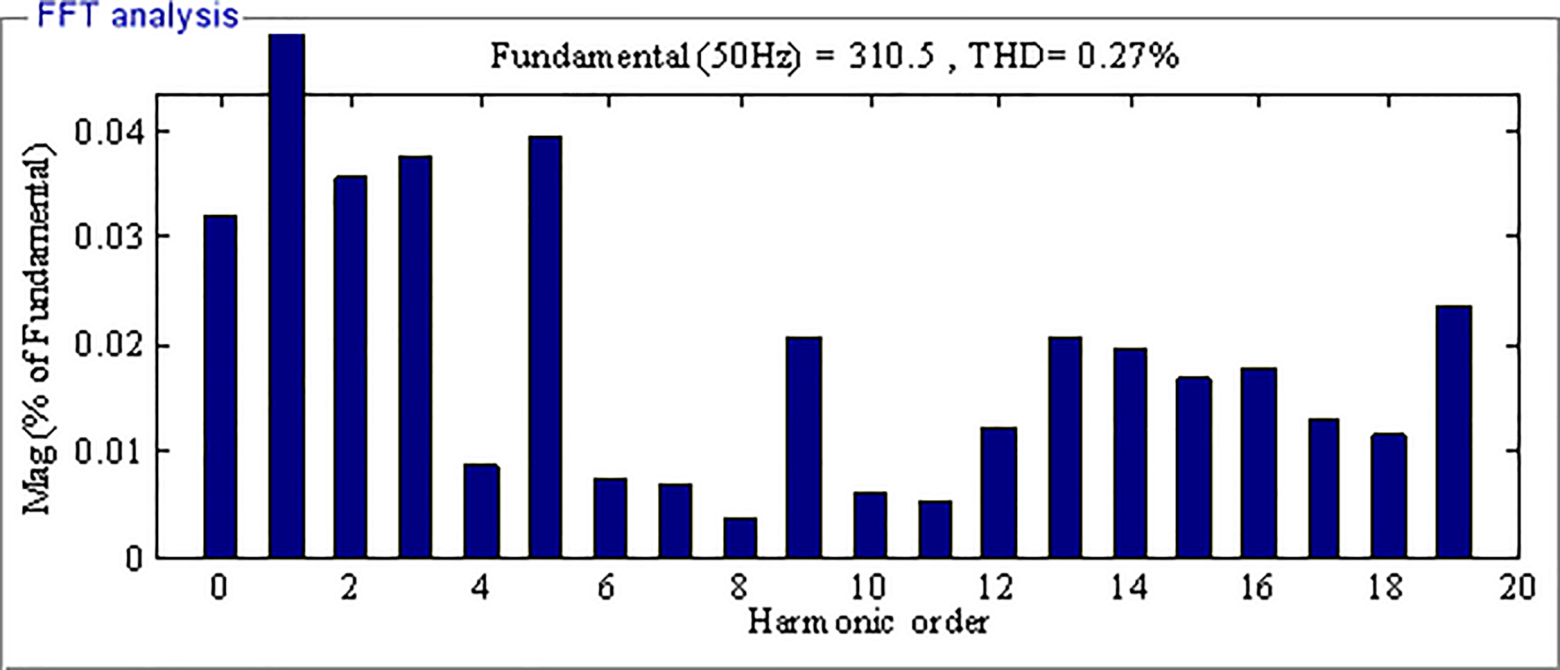
FFT analysis of the output voltage using SMC.

Fig 14 and Fig 15 depict the FFT analysis results of the output voltage using AFSMC and SMC respectively. As can be seen, the THD of the voltage using SMC in steady state is only 0.03%, which is much smaller than that of the output voltage using SMC, indicating the high power quality using the proposed strategy.

In fact, the AFSMC adopts a fuzzy system to estimate the upper bound of the uncertainties of the inverter system which enhances its robustness for the nonlinearities can be adaptively compensated. SMC achieve its performance by employing a switching term to compensate the impact of uncertain disturbances, and the switching gain is chosen generally large for there is no prior knowledge about the disturbance, as a result there may be large chattering in SMC. It can be concluded that the AFSMC has better tracking performance than SMC with smaller tracking error, and higher power quality.

## Conclusion

In this paper, the MPPT and the DC-AC inverter controlling are accomplished on a small scale two-stage single-phrase PV grid-connected inverter system. An INC scheme with an adaptive step size is adopted to track the maximum power point by controlling the duty cycle of the controllable power switch of the boost DC-DC converter. An adaptive fuzzy sliding mode controller with an integral sliding surface is developed for the grid-connected inverter, a fuzzy system is used to approach the upper bound of the system nonlinearities. The simulation results of MPPT indicate that the proposed INC strategy can be adapted to the environment variations and track the MPP quickly. The performance of voltage tracking shows that the inverter is not sensitive to the environmental variations, indicating the robustness of the proposed AFSMC scheme. Comparison results with SMC verify the superiority of the proposed AFSMC strategy.

## Supporting information

S1 File Nomenclature. (PDF)
