# Supplementary material for: Adaptive fuzzy sliding control of single-phase PV grid-connected inverter
Source: PLoS One. 2017 Aug 10;12(8):e0182916. doi: 10.1371/journal.pone.0182916 (PMC5552357; doi:10.1371/journal.pone.0182916)
Supplement: S1 File — (PDF) [file pone.0182916.s001.pdf]

Nomenclature

S_b	Power switch of the DC-DC converter
L_{pv}	Inductor of the DC-DC converter
C_{dc}	Capacitor of the DC-DC converter
D_{pv}	Diode of the DC-DC converter
S_1, S_2, S_3, S_4	Power switch of the inverter
L_{ac}	Capacitor of the inverter at the AC side
C_{ac}	Inductor of the inverter at the AC side,
R_L	Load at the grid side.
C_{pv}	Capacitor at PV side
U_{pv}	PV output voltage
I_{pv}	PV output current
λ	Parameter of INC strategy
D_b	Duty cycle of S_b
k_1, k_2	Sliding surface parameters
u_{ac}	Inverter output voltage
u_{ref}	Grid reference voltage
D	Duty cycle of S_1 and S_4
u_{dc}	DC side voltage of the inverter
g	Uncertain disturbances
g_E	Upper bound of uncertain disturbances
